# Counties with Lower Insurance Coverage and Housing Problems Are Associated with Both Slower Vaccine Rollout and Higher COVID-19 Incidence

**DOI:** 10.3390/vaccines9090973

**Published:** 2021-08-31

**Authors:** Gregory Donadio, Mayank Choudhary, Emily Lindemer, Colin Pawlowski, Venky Soundararajan

**Affiliations:** nference, One Main Street, East Arcade, Cambridge, MA 02142, USA; greg@nference.net (G.D.); mayank@nference.net (M.C.); elindemer@nference.net (E.L.); colin@nference.net (C.P.)

**Keywords:** insurance coverage, vaccination rollout, COVID-19 incidence, socioeconomic factors

## Abstract

Equitable vaccination distribution is a priority for outcompeting the transmission of COVID-19. Here, the impact of demographic, socioeconomic, and environmental factors on county-level vaccination rates and COVID-19 incidence changes is assessed. In particular, using data from 3142 US counties with over 328 million individuals, correlations were computed between cumulative vaccination rate and change in COVID-19 incidence from 1 December 2020 to 6 June 2021, with 44 different demographic, environmental, and socioeconomic factors. This correlation analysis was also performed using multivariate linear regression to adjust for age as a potential confounding variable. These correlation analyses demonstrated that counties with high levels of uninsured individuals have significantly lower COVID-19 vaccination rates (Spearman correlation: −0.460, *p*-value: <0.001). In addition, severe housing problems and high housing costs were strongly correlated with increased COVID-19 incidence (Spearman correlations: 0.335, 0.314, *p*-values: <0.001, <0.001). This study shows that socioeconomic factors are strongly correlated to both COVID-19 vaccination rates and incidence rates, underscoring the need to improve COVID-19 vaccination campaigns in marginalized communities.

## 1. Introduction

Since it was first declared a global pandemic by the World Health Organization (WHO) on 11 March 2020 [1], the novel coronavirus disease 2019 (COVID-19) has developed into the worst pandemic in over 100 years [2]. As of 13 August 2021, there have been more than 200 million cases of COVID-19 reported worldwide, including more than 4.3 million reported deaths [3]. In the United States alone, there have been over 36 million reported COVID-19 cases and 600,000 reported deaths [4]. This has resulted in the deepest global economic recession since World War 2 [5]. 

To combat this deadly pandemic, companies and researchers around the world have been racing to develop treatments [6] and vaccines [7], and national governments have been working to obtain access to vaccines and rapidly administer them to their populations. There are currently seven vaccines for COVID-19 approved for use by the WHO, which are manufactured by Pfizer/BioNTech, Moderna, Johnson & Johnson, Oxford/AstraZeneca, Serum Institute of India, Sinopharm, and Sinovac [8]. To date, 30.8% of the world’s population has received at least one dose of a COVID-19 vaccine, and 16.1% are fully vaccinated [9]. 

The COVID-19 vaccine rollout in the United States has been among one of the fastest in the world [10]. However, this rapid vaccine rollout has not benefited all Americans equally, and the vaccination rate in some marginalized communities has lagged significantly behind the average [11]. Social determinants of health (SDoH) and aspects of an individual’s life that occur “outside of the four walls of healthcare” have a tremendous impact on actual health status [12,13]. A recent study by the CDC showed that vaccine coverage is lower in counties with high social vulnerability based upon socioeconomic indicators (poverty, unemployment, low income, no high school diploma) [14]. This study did not, however, assess the interplay between these factors and new COVID-19 incidence rates. In addition, another recent analysis of 580 US counties found that the change in COVID-19 incidence from 1 December 2020 to 1 March 2021 is significantly correlated with cumulative vaccination rate through 1 March 2021 [15]. Outside of the US, researchers have found significant correlations between socioeconomic status and vaccination acceptance rates in Israel [16], and vaccine hesitancy is a worldwide issue [17]. However, it remains unclear whether disparities in vaccine rollout and associated COVID-19 infection rate fluctuations have been driven by some specific socioeconomic and population health factors. 

The objective of this study is to determine which socioeconomic and environmental factors at the county level affect vaccination and COVID-19 incidence in the US. The following research questions were considered:

(1) Which county-level socioeconomic factors are most strongly correlated with low vaccination rates and high COVID-19 incidence?

(2) Which county-level socioeconomic factors are most strongly correlated with low vaccination rates and high COVID-19 incidence, after adjusting for age as a confounding factor?

(3) What are the characteristics of counties with the lowest vaccination rates?

To address these research questions, publicly available data on US county-level vaccination rates and COVID-19 incidence rates were considered, along with a large dataset of 44 county-level socioeconomic factors. Pairwise correlation analysis between each of the socioeconomic factors with vaccination rates and COVID-19 incidence rates was performed, along with an age-adjusted pairwise correlation analysis to account for age as a confounding factor in the vaccine rollout. Enrichment analysis was performed to determine the socioeconomic factors that differentiate the counties in the top and bottom quartiles of vaccination rate. Finally, multivariate analysis was performed to determine which socioeconomic factors are heavily correlated with each other and which are significant predictors of vaccination rate in a model, controlling for all of the other socioeconomic factors. 

## 2. Materials and Methods

The study analyzed 3142 counties and county equivalents in the United States that have cumulative vaccination data available through 6 June 2021. These counties include over 328 million individuals from all 50 states and the District of Columbia. For each county, the *vaccination rate* was defined as the percentage of individuals in the county with at least one dose of an FDA-authorized COVID-19 vaccine as of 6 June 2021. In addition, county-level COVID-19 incidence data were obtained from the CDC COVID Data Tracker [4]. The *change in COVID-19 incidence* is defined as the 7-day rolling average COVID-19 incidence rate on 6 June 2021 minus the 7-day rolling average COVID-19 incidence rate on 1 December 2020, where the COVID-19 incidence rate is the number of new COVID-19 cases reported in the county per 100,000 individuals. Additional analysis was performed using multivariate linear regression to adjust for age as a potential confounding variable.

Demographic and socioeconomic data for each county were obtained from the 2020 County Health Rankings [18] resource provided by the County Health Rankings & Roadmaps program at the University of Wisconsin Population Health Institute. A data completeness threshold of 70% was set and redundant variables were filtered out, resulting in 44 out of a total of 131 variables from the 2020 County Health Rankings. Most of the variables with limited data availability were race-specific variables for minority populations (e.g., number of firearm fatalities—Black, motor vehicle crash deaths—Hispanic), so these could not be included in the analysis. A complete list of demographic and socioeconomic variables with data available is included in Table 1. For each of the 44 county-level features, Spearman rank correlations were computed between: (1) the feature of interest and county-level *vaccination rate*, and (2) the feature of interest and county-level *change in COVID-19 incidence*. Spearman rank correlations and corresponding *p*-values were computed using the SciPy package (version 1.6.0) [19] in Python. These plots were created using Python’s Matplotlib package (version 3.3.4) [20]. 

In order to evaluate the effect of each standardized county-level feature on vaccine coverage after controlling for age as a confounding factor, a linear regression model was implemented to get standardized coefficients, along with their 95% confidence intervals. The *p*-values were corrected using the Benjamini–Hochberg procedure [21] to avoid Type I error.

Next, each county was grouped into quartiles based on percent vaccinated through 6 June 2021. For a select number of county-level features, rates were computed in the top and bottom quartiles, and relative risks and Fisher exact test *p*-values were reported. For the relative risk values, 95% confidence intervals were computed using a delta-method approximation [22]. 

To analyze the relationship between each pair of features, a correlation matrix was calculated using the Spearman method, and the results are presented in a heatmap. In addition, principal component analysis was used to explore multivariate relationships in the dataset. For this analysis, principal components were computed using features standardized around the mean, and missing values were filled in using the expectation–maximization algorithm. Afterwards, Spearman correlations between each feature and each of the principal components were computed. The results are presented in a heatmap. 

Finally, multivariate regression analysis was performed in order to determine how much each feature influences vaccination rates when all other features are kept constant. In particular, a logistic regression model with L1 regularization was trained to predict whether a county was in the top or bottom quartile based upon vaccination rate, using all of the 44 socioeconomic factors as predictors. The logistic regression model was implemented using the statsmodels (version 0.12.2) package in Python, and the optimal value of the L1 penalty term hyperparameter was computed using cross-validation. 

## 3. Results

**Figure 1:**
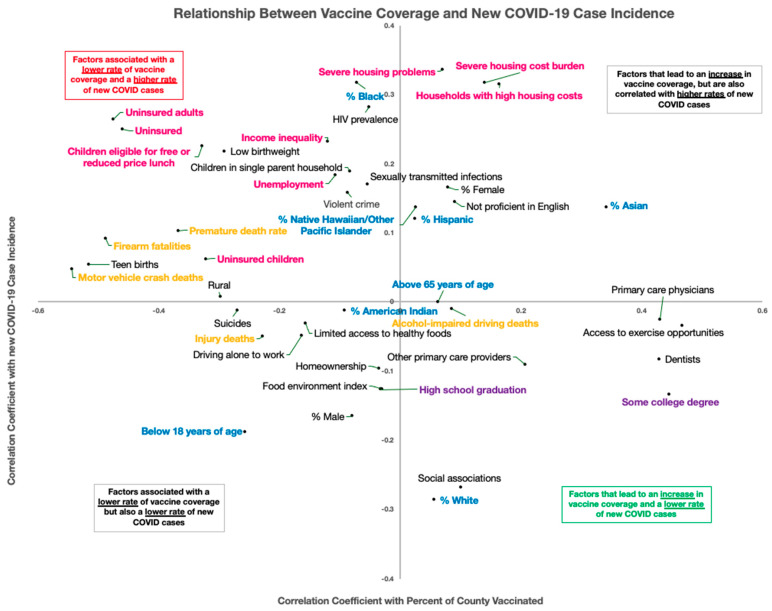
**Relationship between county-level vaccine coverage and change in COVID-19 incidence rate for county-level features.** The *x*-axis shows the Spearman rank correlation between the county-level feature and cumulative vaccination rate (percent of individuals in the county with 1+ vaccine dose as of 6 June 2021). The *y*-axis shows the Spearman rank correlation between the county-level feature and the change in COVID-19 incidence rate (defined as the 7-day rolling average COVID-19 incidence rate on 6 June 2021 minus the 7-day rolling average COVID-19 incidence rate on 1 December 2020). Factors are only shown here if their Spearman coefficient is greater than 0.1 along at least one dimension. Factors in pink are related to housing and income, factors in orange are related to environmental risk, factors in purple are related to education level, and factors in blue are related to race.

Results from the correlation analyses are synthesized together in Figure 1. Each socioeconomic variable is plotted to show the strength of its relationship with county-level vaccine coverage (*x*-axis) and county-level new COVID-19 incidence (*y*-axis). The upper left quadrant contains variables that are associated with both increased incidence and poor vaccine coverage, and the bottom right quadrant contains variables that are associated with decreased incidence and better vaccine coverage. Intervariable correlations are shown in Figure S1.

### 3.1. Insurance Coverage and Vaccination Rates

**Figure 2:**
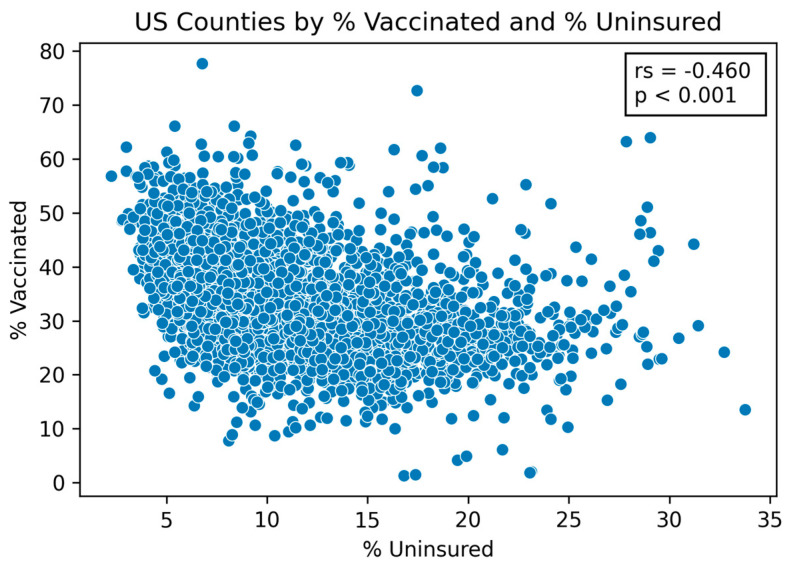
**US counties by percentage vaccinated and percentage uninsured.** The figure shows the correlation between the percent of the population that is uninsured and vaccine coverage (r_s_ = Spearman correlation coefficient, *p* = significance level).

**Figure 3:**
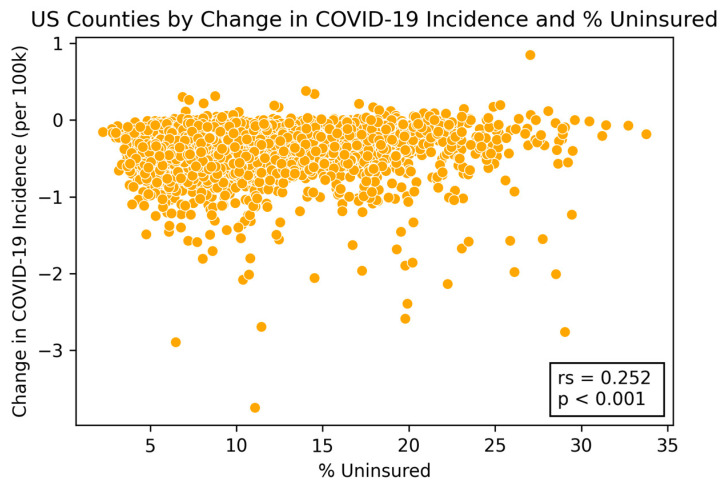
**US counties by change in COVID-19 incidence and high housing costs.** The figure shows the correlation between the percent of households with high housing costs and new COVID-19 case incidence (r_s_ = Spearman correlation coefficient, *p* = significance level).

Factors related to housing and income were shown to have strong correlations with lower vaccination rates and higher incidence cases compared to the national average. Two of these factors, the percentage of uninsured individuals and the percentage of children eligible for free lunch, were both significantly negatively correlated with the percentage of vaccinated individuals (Spearman correlation: −0.460, *p*-value: <0.001; Spearman correlation: −0.328, *p*-value: <0.001) (see Figure 2 and Table 1). The relationship between these two factors and the incidence change in COVID-19 cases from 1 December 2020 to 6 June 2021 were significantly positive (uninsured individuals—Spearman correlation: 0.252, *p*-value: <0.001; children eligible for free lunch—Spearman correlation: 0.225, *p*-value: <0.001) (see Figure 3 and Table 1).

A county’s percentage of motor vehicle crash deaths and teen births were both significantly correlated with the percentage of the county that had been vaccinated (Spearman correlation: −0.543, *p*-value: <0.001; Spearman correlation: −0.515, *p*-value: <0.001) (see Table 1). However, the relationships between these two factors and the incidence change in COVID-19 cases from 1 December 2020 to 6 June 2021 (Spearman correlation: −0.047, *p*-value: 0.71; Spearman correlation: 0.054, *p*-value: 0.70) were insignificant.

Similar results were seen with the percentage of firearm fatalities. This factor was significantly correlated with the percentage of the county that had been vaccinated (Spearman correlation: −0.487, *p*-value: <0.001), but the factor’s relationship with the incidence change in COVID-19 cases from 1 December 2020 to 6 June 2021 was not as strong (Spearman correlation: 0.091, *p*-value: 0.001).

There was a slight negative correlation between unemployment level and percentage of individuals that had been vaccinated (Spearman correlation: −0.107, *p*-value: <0.001) and a positive correlation between unemployment levels and new cases (Spearman correlation: 0.182, *p*-value: <0.001). There was no significant correlation, however, between unemployment rate and insurance coverage (Spearman correlation: 0.003, *p*-value: 0.85), indicating that these findings with insurance coverage are not driven by unemployment.

**Figure 4:**
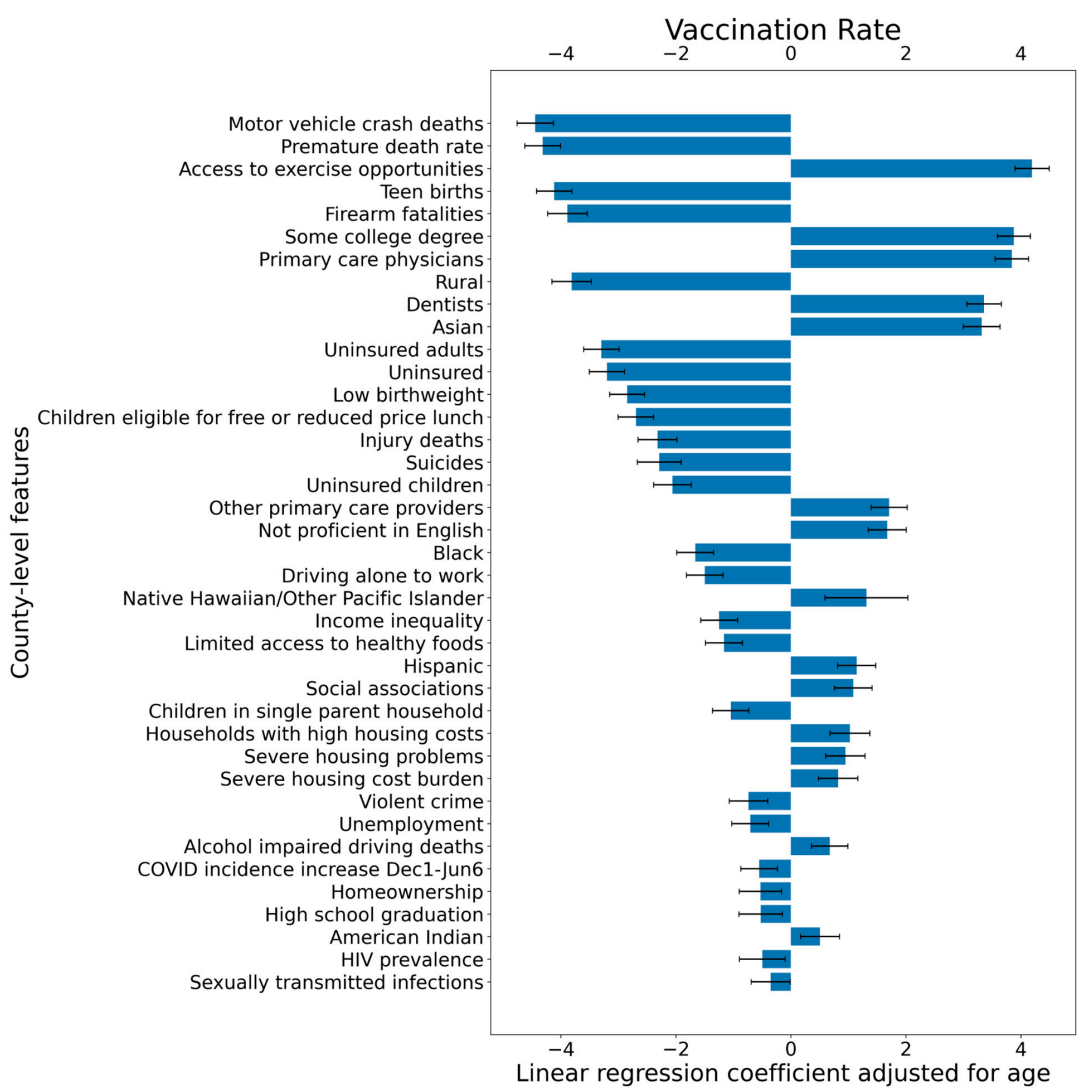
**Linear regression coefficients showing the relationship between vaccination rates and county-level features.** County-level vaccination percentage is defined as 100% × (the number of individuals in the county with at least one COVID-19 vaccine dose as of 6 June 2021)/(total population of the county). An increase in a county-level feature of one standard deviation corresponds to a change in the county-level vaccination rate in percentage after controlling for age as a confounding variable. For each coefficient, error bars corresponding to the 95% confidence intervals are shown as well.

Table 2 shows estimated coefficients of a linear regression for each standardized county-level feature, indicating their effect on vaccination rates after adjusting for age as a confounding variable when that feature is increased by one standard deviation. These regression coefficients, along with their corresponding 95% confidence intervals, are visualized in Figure 4 as well. County-level features related to poverty, such as motor vehicle crash deaths (regression coefficient: −4.4, *p*-value: <0.001), premature death rate (regression coefficient: −4.3, *p*-value: <0.001), teen births (regression coefficient: −4.1, *p*-value: <0.001), firearm fatalities (regression coefficient: -3.9, *p*-value: <0.001), rural (regression coefficient: −3.8, *p*-value: <0.001), and uninsured prevalence in adults (regression coefficient: −3.3, *p*-value: <0.001), show the strongest negative associations. Features such as access to exercise opportunity (regression coefficient: 4.2, *p*-value: <0.001) and individuals with some college degree (regression coefficient: 3.9, *p*-value: <0.001), access to primary care physicians (regression coefficient: 3.8, *p*-value: <0.001) and dentists (regression coefficient: 3.3, *p*-value: <0.001), and counties with a high number of individuals identified as Asian (regression coefficient: 3.3, *p*-value: <0.001) had the strongest positive correlation among all demographic features.

Table 3 demonstrates that the relative risk related to the percent of adults living in rural counties (0.236, top quartile rate: 11.579, bottom quartile rate: 49.146) in the population was the lowest. Relative risks related to socioeconomic county-level features, including teen births (0.467, top quartile rate: 1.669%, bottom quartile rate: 3.573%), firearm fatalities (0.479, top quartile rate: 0.008%, bottom quartile rate: 0.018%), disconnected youth (0.574, top quartile rate: 5.603%, bottom quartile rate: 9.579%), uninsured adults (0.577, top quartile rate: 9.579%, bottom quartile rate: 16.614%), and homicides (0.577, top quartile rate: 0.004%, bottom quartile rate: 0.008%) in the top 25% counties were the lowest when compared with counties in the bottom quartile.

Relative risks related to socioeconomic features, such as access to primary care physicians (1.963, top quartile rate: 0.09%, bottom quartile rate: 0.046%) and dentists (1.943, top quartile rate: 0.082%, bottom quartile rate: 0.042%), HIV prevalence (1.66, top quartile rate: 0.37%, bottom quartile rate: 0.223%), access to exercise opportunities (1.547, top quartile rate: 91.68%, bottom quartile rate: 59.287%), and severe housing cost burdens (1.328, top quartile rate: 15.826%, bottom quartile rate: 11.921%), had the highest relative risks for individuals in the top 25% counties compared with individuals in the bottom 25% vaccinated county.

For racial/ethnic groups, counties with a greater number of Asian (6.461, top quartile rate: 8.999%, bottom quartile rate: 1.393%), Hawaiian/Pacific Islander (1.886, top quartile rate: 0.301%, bottom quartile rate: 0.16%), and Hispanic (1.758, top quartile rate: 18.547%, bottom quartile rate: 10.287%) individuals had higher relative risks, whereas counties with greater numbers of American Indian (0.454, top quartile rate: 1.081%, bottom quartile rate: 2.381%), and Black (0.649, top quartile rate: 10.418%, bottom quartile rate: 16.049%) individuals had lower relative risks. 

Table 3 demonstrates that the relative risk related to the percent of uninsured adults in the population is 0.577, where counties in the top quartile of vaccine coverage have 9.579% uninsured adults and those in the bottom quartile have 16.614%. This translates to counties in the top quartile of vaccination coverage having a 42% lower uninsured population compared to those in the bottom quartile. 

Table 4 looks at the top and bottom quartiles of counties, ranked by percent of the county that is uninsured. On average, counties in the bottom quartile have an uninsurance rate of 18.7%. Overall, counties with lower insurance coverage rates tended to be more rural, have higher populations of minorities, and have higher populations of young people. The states contributing to these counties the most are Texas, Georgia, Oklahoma, Mississippi, and Florida. The top 25 US counties with the highest proportions of uninsured individuals are detailed in Table S1.

### 3.2. Housing Problems and COVID-19 Incidence 

The strongest correlates of COVID-19 incidence in 2021 were the percent of households in a county with high housing costs and the percent of households with severe housing problems (Spearman correlations: 0.314, 0.335, *p*-values: <0.001, <0.001). In addition, other housing problem factors had some of the most positive correlations with COVID-19 incidences compared to all of the included variables. These housing factors were rates of households with high housing costs, income inequality, children eligible for free or reduced-price lunch, and unemployment.

### 3.3. Environmental Risk Factors, Education, and Vaccination Rates

Annual incidence of motor vehicle crash deaths and incidence of firearm fatalities were both negatively correlated with vaccine coverage and positively correlated with new COVID-19 case incidence (Table 1). Violent crime was also negatively associated with vaccine coverage. On the other hand, access to exercise opportunities was positively correlated with vaccine coverage and COVID-19 incidence rates. In addition, college completion rates by 2020 were positively correlated with vaccine coverage; however, this variable was only weakly negatively correlated with COVID-19 incidence rates. Finally, social association ranking, as reflected by the number of civic organizations in the county, was weakly positively correlated with vaccine coverage and strongly negatively correlated with COVID-19 incidence rates (Table 1).

### 3.4. Principal Component Analysis

From the principal component analysis, factors associated with poverty and environmental risks had the strongest negative correlations with the first principal component, which accounts for the greatest variation in the data. This includes variables such as teen births, children eligible for free or reduced-price lunch, uninsured population, annual incidence of motor vehicle crash deaths, incidence of firearm fatalities, and low birthweight (Figure S2). In contrast, factors that are highly associated with affluent communities had strong positive correlations with the first principal component. This includes variables such as college degree, access to exercise opportunities, dentists, primary care physicians, and high school graduation (Figure S2). 

The second principal component, which captures the second highest variation in the data, was strongly negatively correlated with high housing costs, severe housing cost burden, and severe housing problems. On the other hand, the second principal component was also strongly positively correlated with rural counties and homeownership (Figure S2). These results suggest that factors related to housing problems contribute to a significant source of variation in the dataset, and these factors are distinct from the number of uninsured individuals, number of primary care physicians, and other socioeconomic factors that are strongly correlated with the first principal component. 

### 3.5. Logistic Regression Analysis

Figure S3 shows the magnitude of coefficients in the L1 logistic regression model to predict which counties are in the top vs. bottom quartile based upon vaccination rate. The logistic regression coefficients, 95% confidence intervals for the logistic regression coefficients, and associated *p*-values are also presented in Table S2. In this model, an increase in a county-level feature of one standard deviation corresponds to the amount of increase in the predicted log odds of counties with most vaccination coverage, holding all other features constant. Among the 44 socioeconomic features considered, 31 features were selected by the model to have non-zero coefficients. The feature representing uninsured adults per 100,000 people (odds ratio: 0.30, 95% CI: (0.23, 0.41): *p*-value: <0.001) has the strongest negative correlation with vaccination rate, with all other variables held constant. 

## 4. Discussion

At a high level, this study highlights the fact that socioeconomic factors are highly correlated with county-level vaccination rates and COVID-19 incidence rates. In particular, the proportion of uninsured individuals was observed to be significantly negatively correlated with vaccination rates and positively correlated with COVID-19 incidence rates, and the proportion of individuals with housing problems was observed to be significantly correlated with COVID-19 incidence rates. Prior studies in the United States [14] and Israel [16] have found that socioeconomic vulnerability is linked with lower vaccination rates; however, these studies focus on the concept of a socioeconomic vulnerability index more broadly, rather than on individual socioeconomic factors. Another recent US-based study found that vaccination rates are strongly correlated with housing problems [14,23]; however, this analysis did not consider COVID-19 incidence rates as an additional outcome measure. Furthermore, this current study is novel because it includes a large number of socioeconomic factors in the analysis. 

Despite the US government’s financial sponsorship of the COVID-19 vaccine [24], this study shows a strong relationship between county-level health insurance status, percent of the county that has been vaccinated, and the incidence of new cases since the beginning of 2021. Of all variables studied, insurance coverage was one of the most strongly associated with vaccination coverage. This may be due to the fact that many individuals receive information about general health, and also about their vaccine eligibility status from their primary care provider [25]. In particular, individuals without health insurance may receive less information about their eligibility for COVID-19 vaccines and less information about the precautions that they can take to reduce their risk of COVID-19 infection in general. Direct messaging from the government to inform individuals that they are eligible for the vaccine regardless of insurance status may have a significant impact on both vaccine coverage and new case incidence rates.

When assessing socioeconomic factors related to COVID-19 incidence in 2021, the strongest relationships were with factors relating to severe housing problems. According to County Health Rankings, this is defined as the percentage of households with at least one of the following four housing problems: overcrowding, high housing costs, lack of kitchen facilities, or lack of plumbing facilities [26]. Given that one of the most effective ways to avoid the spread of COVID-19 is social distancing, the findings related to housing problems and new spread of disease are expected. Along similar lines, prior studies have also shown positive correlations between population density and case incidence [27]. 

Factors pertaining to race, wealth, housing, and education status are tightly intertwined when it comes to healthcare [28,29]. To this end, it is not surprising that similar trends were seen with lower education, poorer housing status, income inequality, and racial minorities that move in the same direction in the analyses. All of these factors show some relationship with poorer vaccine coverage and higher recent incidence rates. This work highlights that there are also environmental risk factors that fall into the same pattern. Many factors that fall into the bottom-right quadrant of Figure 1, the quadrant with the most favorable outcomes, pertain to having a higher education, a higher-paying career, general quality of life, social connectivity, and being white. For the most part, factors that fall into the top-right and bottom-left quadrants with mixed outcomes are educational factors signifying a mid-range level of education, or pertain to age-related factors that directly impact vaccine eligibility, such as being under 18 years old. 

There are several limitations of this study. First, only 44 of the original 131 variables were able to be utilized due to limitations with data availability. Many variables that were lacking in complete data were those at the intersection of racial minority status and other socioeconomic factors, such as homicide rates within specific racial segments. Specifically, 52 of the 63 incomplete variables were specific to racial minority groups, and all data variables with less than 35% completeness were specific to racial minority groups. Had this been available, the study may have been able to parse out more specific relationships of COVID-19, vaccination coverage, and racial minorities. Additionally, one of the challenges in assessing both vaccine coverage, as well as new incidence rates, is in the diversity of state roll-out plans in terms of timeline and eligibility criteria. A future retrospective analysis comparing individual states is an important next step to be taken when more data have been collected across the nation.

There are multiple promising areas for future research. Targeted questionnaires and patient focus groups could be used to determine the reasons that patients without insurance coverage are vaccinated at lower rates. In addition, this line of research could be used to come up with interventions and public policy to make vaccine distribution more equitable, especially among the uninsured population. Similar follow-up research focusing on populations with housing problems could be used to determine the reasons that COVID-19 incidence rates are elevated in this population and which interventions may be most effective. 

## 5. Conclusions

The main implications of this research are that socioeconomic factors are significant drivers of vaccination rates and COVID-19 incidence rates. In particular, the results show that populations without health insurance and with housing problems are particularly vulnerable, highlighting the need to progress COVID-19 vaccination campaigns for these groups. These findings reinforce and build upon what is known about the vast socioeconomic disparities that are still ongoing in the US surrounding the COVID-19 pandemic. 

It was shown that the most significant factors associated with low vaccination rates at the county level are those related to poverty and environmental safety, such as uninsurance prevalence, teen births, firearm fatalities, and motor vehicle crash deaths. On the other hand, the most significant protective factors are related to college education, social connectivity, and high prevalence of medical professionals. Among the factors associated with high COVID-19 incidence rates, severe housing problems and high costs of housing were found to have the strongest correlations. Taken together, these findings suggest that addressing socioeconomic inequalities will be important in order to increase vaccine coverage across the United States and to reduce future COVID-19 surges in counties with socioeconomically vulnerable populations. 

## Figures and Tables

**Table 1:** **Spearman rank correlations for county-level features with vaccination rates and change in COVID-19 incidence rates.** The county-level vaccination rate is defined as the percentage of individuals in the county with at least one COVID-19 vaccine dose as of 6 June 2021. The county-level COVID-19 incidence rate increase is defined as the 7-day rolling average COVID-19 incidence rate (number of new COVID-19 cases/total population) in the county on 6 June 2021 minus the 7-day rolling average COVID-19 incidence rate in the county on 1 December 2020. For each county-level feature, we show the Spearman rank correlation coefficients for the feature vs. vaccination rate, and the feature vs. COVID-19 incidence rate increase, along with the associated *p*-values. Rows are sorted by correlation with vaccination rate.

County-Level Feature	Vaccination Rate Correlation	Vaccination Rate *p*-Value	COVID-19 Incidence Rate Increase Correlation	COVID-19 Incidence Rate Increase *p*-Value
Motor vehicle crash deaths	−0.543	<0.001	0.047	0.712
Teen births	−0.515	<0.001	0.054	0.706
Firearm fatalities	−0.487	<0.001	0.091	0.001
Uninsured adults	−0.475	<0.001	0.264	<0.001
Uninsured	−0.460	<0.001	0.252	<0.001
Premature death rate	−0.366	<0.001	0.102	<0.001
Children eligible for free or reduced-price lunch	−0.328	<0.001	0.225	<0.001
Uninsured children	−0.321	<0.001	0.062	0.890
Rural	−0.297	<0.001	0.007	0.062
Low birthweight	−0.290	<0.001	0.217	<0.001
Suicides	−0.270	<0.001	−0.013	0.003
Below 18 years of age	−0.257	<0.001	−0.189	<0.001
Injury deaths	−0.227	<0.001	−0.049	<0.001
Driving alone to work	−0.163	<0.001	−0.049	0.947
Limited access to healthy foods	−0.157	<0.001	−0.031	<0.001
Income inequality	−0.119	<0.001	0.231	<0.001
Unemployment	−0.107	<0.001	0.183	<0.001
American Indian	−0.092	0.027	−0.012	<0.001
Violent crime	−0.086	<0.001	0.157	<0.001
Children in single-parent household	−0.083	<0.001	0.188	<0.001
Male	−0.079	<0.001	−0.165	<0.001
Black	−0.071	<0.001	0.317	<0.001
Sexually transmitted infections	−0.054	<0.001	0.169	<0.001
HIV prevalence	−0.051	0.971	0.281	<0.001
Homeownership	−0.035	0.015	−0.096	<0.001
High school graduation	−0.032	0.044	−0.126	<0.001
Food environment index	−0.030	0.002	−0.127	<0.001
Hispanic	0.024	0.062	0.119	0.759
Native Hawaiian/Other Pacific Islander	0.025	0.002	0.136	<0.001
White	0.055	0.060	−0.285	<0.001
Above 65 years of age	0.062	<0.001	0.000	0.745
Severe housing problems	0.071	<0.001	0.335	<0.001
Female	0.079	<0.001	0.165	<0.001
Alcohol-impaired driving deaths	0.086	<0.001	−0.011	0.022
Not proficient in English	0.090	<0.001	0.144	0.086
Social associations	0.100	<0.001	−0.268	<0.001
Severe housing cost burden	0.139	<0.001	0.316	<0.001
Households with high housing costs	0.164	<0.001	0.314	<0.001
Other primary care providers	0.208	<0.001	−0.091	<0.001
Asian	0.342	<0.001	0.136	<0.001
Dentists	0.429	<0.001	−0.083	0.333
Primary care physicians	0.430	<0.001	−0.026	0.37
Some college degree	0.446	<0.001	−0.134	<0.001
Access to exercise opportunities	0.468	<0.001	−0.035	0.575

**Table 2:** **Linear regression coefficients between vaccination rates and county-level features after adjusting for age as a confounding variable.** County-level vaccination percentage is defined as 100% × (the number of individuals in the county with at least one COVID-19 vaccine dose as of 6 June 2021)/(total population of the county). For each county-level feature, linear regression models were fit to predict the outcome variable using the county-level feature and age as the independent variables. For each model, linear regression coefficients and their 95% confidence intervals are shown, along with the associated *p*-values. Rows are sorted by correlation with vaccination. For example, according to the first linear model, an increase in motor vehicle crash deaths of one standard deviation corresponds to a change in the county-level vaccination rate of −4.4% (95% CI: (−4.8%, −4.1%)).

County-Level Feature	Coefficient (95% CI)	*p*-Value
Motor vehicle crash deaths	−4.4 (−4.8, −4.1)	<0.001
Premature death rate	−4.3 (−4.6, −4.0)	<0.001
Teen births	−4.1 (−4.4, −3.8)	<0.001
Firearm fatalities	−3.9(−4.2, −3.5)	<0.001
Rural	−3.8 (−4.2, −3.5)	<0.001
Uninsured adults	−3.3 (−3.6, −3.0)	<0.001
Uninsured	−3.2 (−3.5, −2.9)	<0.001
Low birthweight	−2.8 (−3.2, −2.5)	<0.001
Children eligible for free or reduced-price lunch	−2.7 (−3.0, −2.4)	<0.001
Injury deaths	−2.3 (−2.7, −2.0)	<0.001
Suicides	−2.3 (−2.7, −1.9)	<0.001
Uninsured children	−2.1 (−2.4, −1.7)	<0.001
Black	−1.7 (−2.0, −1.3)	<0.001
Driving alone to work	−1.5 (−1.8, −1.2)	<0.001
Income inequality	−1.2 (−1.6, −0.9)	<0.001
Limited access to healthy foods	−1.2 (−1.5, −0.8)	<0.001
Children in single-parent household	−1.0 (−1.4, −0.7)	<0.001
Violent crime	−0.7 (−1.1, −0.4)	<0.001
Unemployment	−0.7 (−1.0, −0.3)	<0.001
COVID-19 incidence increase Dec1-Jun6	−0.5 (−0.8, −0.2)	0.001
Homeownership	−0.5 (−0.9, −1.6)	0.005
High school graduation	−0.5 (−0.9, −0.1)	0.007
HIV prevalence	−0.5 (−0.9, −0.1)	0.015
Sexually transmitted infections	−0.4 (−0.7, 0.0)	0.044
American Indian	0.5 (0.2, 0.8)	0.004
Alcohol-impaired driving deaths	0.7 (0.4, 1.0)	<0.001
Severe housing cost burden	0.8 (0.5, 1.2)	<0.001
Severe housing problems	0.9 (0.6, 1.3)	<0.001
Households with high housing costs	1.0 (0.7, 1.4)	<0.001
Social associations	1.1 (0.8, 1.4)	<0.001
Hispanic	1.1 (0.8, 1.5)	<0.001
Native Hawaiian/Other Pacific Islander	1.3 (0.6, 2.0)	<0.001
Not proficient in English	1.7 (1.3, 2.0)	<0.001
Other primary care providers	1.7 (1.4, 2.0)	<0.001
Asian	3.3 (3.0, 3.6)	<0.001
Dentists	3.3 (3.1, 3.6)	<0.001
Primary care physicians	3.8 (3.6, 4.1)	<0.001
Some college degree	3.9 (3.6, 4.2)	<0.001
Access to exercise opportunities	4.2 (3.9, 4.5)	<0.001

**Table 3:** **Comparison of county-level features in the top and bottom quartiles of vaccinated counties.** The county-level vaccination rate is defined as the percentage of individuals in the county with at least one COVID-19 vaccine dose as of 6 June 2021. Counties in the top quartile have vaccination rates greater than or equal to 39.12%, and counties in the bottom quartile have vaccination rates less than or equal to 26.58%. Rows have been sorted by relative risk in increasing order.

County-Level Feature	Rate in Top Quartile of Vaccinated Counties	Rate in Bottom Quartile of Vaccinated Counties	Relative Risk (95% CI)	Fisher exact Test *p*-Value
Rural	11.579	49.146	0.236 (0.235, 0.236)	<0.001
American Indian	1.081	2.381	0.454 (0.453, 0.455)	<0.001
Teen births	1.669	3.573	0.467 (0.465, 0.47)	<0.001
Firearm fatalities	0.008	0.018	0.479 (0.471, 0.486)	<0.001
Disconnected youth	5.603	9.756	0.574 (0.57, 0.578)	<0.001
Uninsured adults	9.579	16.614	0.577 (0.576, 0.577)	<0.001
Homicides	0.004	0.008	0.577 (0.564, 0.589)	<0.001
Uninsured children	3.806	6.298	0.604 (0.602, 0.606)	<0.001
Child mortality	0.042	0.067	0.628 (0.616, 0.639)	<0.001
Premature age-adjusted mortality	0.356	0.557	0.639 (0.636, 0.641)	<0.001
Premature death	0.356	0.557	0.639 (0.636, 0.641)	<0.001
Black	10.418	16.049	0.649 (0.649, 0.65)	<0.001
Suicides	0.012	0.018	0.655 (0.645, 0.665)	<0.001
Infant mortality	0.499	0.745	0.67 (0.657, 0.683)	<0.001
Injury deaths	0.061	0.084	0.72 (0.715, 0.725)	<0.001
Children eligible for free or reduced-price lunch	46.376	60.69	0.764 (0.763, 0.765)	<0.001
Low birthweight	7.645	8.901	0.859 (0.855, 0.863)	<0.001
Children in single-parent households	30.41	35.053	0.868 (0.866, 0.869)	<0.001
White	59.103	68.091	0.868 (0.868, 0.868)	<0.001
Unemployment	3.712	4.236	0.876 (0.874, 0.879)	<0.001
Driving alone to work	72.949	82.217	0.887 (0.887, 0.888)	<0.001
Below 18 years of age	21.672	23.977	0.904 (0.903, 0.905)	<0.001
Homeownership	62.281	68.647	0.907 (0.907, 0.908)	<0.001
Social associations	0.089	0.097	0.915 (0.903, 0.928)	<0.001
Food environment index	65.801	70.352	0.935 (0.77, 1.136)	0.535
High school graduation	82.987	86.783	0.956 (0.955, 0.958)	<0.001
Above 65 years of age	15.707	16.24	0.967 (0.966, 0.968)	<0.001
Sexually transmitted infections	0.501	0.518	0.967 (0.962, 0.973)	<0.001
Violent crime	0.343	0.346	0.992 (0.985, 0.999)	0.034
Alcohol-impaired driving deaths	28.91	26.936	1.073 (1.048, 1.099)	<0.001
Drug overdose deaths	0.021	0.018	1.131 (1.109, 1.154)	<0.001
Other primary care providers	0.101	0.082	1.227 (1.21, 1.245)	<0.001
Some college	70.847	54.909	1.29 (1.289, 1.291)	<0.001
Severe housing problems	19.154	14.713	1.302 (1.3, 1.304)	<0.001
Severe housing cost burden	15.826	11.921	1.328 (1.325, 1.33)	<0.001
Access to exercise opportunities	91.68	59.267	1.547 (1.546, 1.547)	<0.001
HIV prevalence	0.37	0.223	1.66 (1.643, 1.677)	<0.001
Hispanic	18.547	10.55	1.758 (1.756, 1.76)	<0.001
Hawaiian/Pacific Islander	0.301	0.16	1.886 (1.866, 1.905)	<0.001
Dentists	0.082	0.042	1.943 (1.905, 1.982)	<0.001
Primary care physicians	0.09	0.046	1.963 (1.926, 2.001)	<0.001
Asian	8.999	1.393	6.461 (6.439, 6.483)	<0.001

**Table 4:** **General characteristics of all counties and counties with the highest and lowest levels of insurance coverage.** The first column (Overall) shows the characteristics for all 3087 counties with vaccination data available. The second column (Top 25%) shows the characteristics for counties with the fewest uninsured individuals per capita (≤7.36%). The third column (Bottom 25%) shows the characteristics for counties with the most uninsured individuals per capita (≥14.57%). Information on state, county population, major town/city, cumulative vaccination till date, and increase in COVID-19 incidence as of 12 April 2021 relative to 1 December 2020 is provided for each group of counties. States with at least one county in the bottom 25% based on insurance coverage are highlighted in **red**.

Characteristic	Overall	Counties with the Highest Rates of Insurance	Counties with the Lowest Rates of Insurance
		Coverage	Coverage
		(Top 25%)	(Bottom 25%)
**Number of counties**	3142	786	772
**Insurance coverage**			
Insured	88.50%	94.10%	81.30%
Uninsured	11.50%	5.90%	18.70%
Cumulative vaccination rate (1+ dose) through 12 April 2021	33.20%	39.00%	19.10%
Change in COVID-19 incidence (cases per 100K) from 1 December 2020 to 6 June 2021	−382.4	−439.9	−315.1
**Population**			
Mean	104,468	137,163	70,209
Std. deviation	333,456	257,454	276,432
IQR	(10,902—68,072)	(18,603—128,468)	(7578—41,088)
**County-type**			
Rural	58.60%	49.20%	65.90%
Urban	41.40%	50.80%	34.10%
**State**			
Alabama	Alabama (67)	Alabama (0)	Alabama (3)
Alaska	Alaska (29)	Alaska (0)	Alaska (24)
Arizona	Arizona (15)	Arizona (0)	Arizona (3)
Arkansas	Arkansas (75)	Arkansas (7)	Arkansas (1)
California	California (58)	California (23)	California (0)
Colorado	Colorado (64)	Colorado (6)	Colorado (5)
Connecticut	Connecticut (8)	Connecticut (7)	Connecticut (0)
Delaware	Delaware (3)	Delaware (3)	Delaware (0)
District of Columbia	District of Columbia (1)	District of Columbia (1)	District of Columbia (0)
Florida	Florida (67)	Florida (0)	Florida (45)
Georgia	Georgia (159)	Georgia (0)	Georgia (128)
Hawaii	Hawaii (5)	Hawaii (4)	Hawaii (0)
Idaho	Idaho (44)	Idaho (0)	Idaho (16)
Illinois	Illinois (102)	Illinois (89)	Illinois (0)
Indiana	Indiana (92)	Indiana (11)	Indiana (2)
Iowa	Iowa (99)	Iowa (85)	Iowa (0)
Kansas	Kansas (105)	Kansas (5)	Kansas (21)
Kentucky	Kentucky (120)	Kentucky (90)	Kentucky (0)
Louisiana	Louisiana (64)	Louisiana (4)	Louisiana (0)
Maine	Maine (16)	Maine (0)	Maine (1)
Maryland	Maryland (24)	Maryland (15)	Maryland (0)
Massachusetts	Massachusetts (14)	Massachusetts (14)	Massachusetts (0)
Michigan	Michigan (83)	Michigan (50)	Michigan (0)
Minnesota	Minnesota (87)	Minnesota (76)	Minnesota (0)
Mississippi	Mississippi (82)	Mississippi (0)	Mississippi (58)
Missouri	Missouri (115)	Missouri (2)	Missouri (34)
Montana	Montana (56)	Montana (0)	Montana (12)
Nebraska	Nebraska (93)	Nebraska (8)	Nebraska (14)
Nevada	Nevada (17)	Nevada (0)	Nevada (2)
New Hampshire	New Hampshire (10)	New Hampshire (4)	New Hampshire (0)
New Jersey	New Jersey (21)	New Jersey (8)	New Jersey (0)
New Mexico	New Mexico (33)	New Mexico (1)	New Mexico (1)
New York	New York (62)	New York (58)	New York (0)
North Carolina	North Carolina (100)	North Carolina (0)	North Carolina (30)
North Dakota	North Dakota (53)	North Dakota (5)	North Dakota (6)
Ohio	Ohio (88)	Ohio (55)	Ohio (1)
Oklahoma	Oklahoma (77)	Oklahoma (0)	Oklahoma (71)
Oregon	Oregon (36)	Oregon (6)	Oregon (0)
Pennsylvania	Pennsylvania (67)	Pennsylvania (44)	Pennsylvania (0)
Rhode Island	Rhode Island (5)	Rhode Island (5)	Rhode Island (0)
South Carolina	South Carolina (46)	South Carolina (0)	South Carolina (9)
South Dakota	South Dakota (66)	South Dakota (1)	South Dakota (21)
Tennessee	Tennessee (95)	Tennessee (1)	Tennessee (4)
Texas	Texas (254)	Texas (0)	Texas (242)
Utah	Utah (29)	Utah (2)	Utah (6)
Vermont	Vermont (14)	Vermont (14)	Vermont (0)
Virginia	Virginia (133)	Virginia (5)	Virginia (12)
Washington	Washington (39)	Washington (15)	Washington (1)
West Virginia	West Virginia (55)	West Virginia (18)	West Virginia (0)
Wisconsin	Wisconsin (72)	Wisconsin (44)	Wisconsin (1)
Wyoming	Wyoming (23)	Wyoming (0)	Wyoming (11)
**Age**			
<18 years old	22.10%	21.50%	23.30%
18–64 years old	58.70%	59.60%	57.90%
≥65 years old	19.20%	18.90%	18.90%
**Gender**			
Male	50.10%	49.90%	50.50%
Female	49.90%	50.10%	49.50%
**Race**			
Black	9.00%	4.30%	12.10%
White	75.80%	86.00%	62.20%
Asian	1.50%	2.20%	1.10%
American Indian	2.00%	0.50%	4.80%
**Ethnicity**			
Hispanic	9.80%	5.10%	17.80%
Not Hispanic	90.20%	94.90%	82.20%

## Data Availability

After publication, the data will be made available to others upon reasonable requests to the corresponding authors (colin@nference.net, venky@nference.net). A proposal with a detailed description of study objectives and the statistical analysis plan will be needed for evaluation of the reasonability of requests.

## Supplementary Materials

The following are available online at https://www.mdpi.com/article/10.3390/vaccines9090973/s1, Figure S1: Heatmap of Spearman’s rank correlation for selected county-level features, Figure S2: Correlation matrix between principal components and county-level features, Figure S3: LASSO logistic regression coefficients and confidence intervals, Table S1: Top 25 US Counties ranked by percent of uninsured population, Table S2: LASSO logistic regression coefficients to differentiate top and bottom quartile vaccinated counties.

## References

[1] Cucinotta D., Vanelli M. (2020). WHO Declares COVID-19 a Pandemic. Acta Biomed. Atenei Parm..

[2] Rosenthal M. Fauci: COVID-19 Worst Pandemic in 100 Years. https://www.idse.net/Covid-19/Article/10-20/Fauci--COVID-19-Worst-Pandemic-in-100-Years/60937.

[3] WHO WHO Coronavirus (COVID-19) Dashboard. https://covid19.who.int/table.

[4] CDC (2020). COVID Data Tracker. https://covid.cdc.gov/covid-data-tracker.

[5] Yeyati E.L., Filippini F. (2021). Social and Economic Impact of COVID-19. Brookings [Internet].

[6] Negrut N., Codrean A., Hodisan I., Bungau S., Tit D.M., Marin R., Behl T., Banica F., Diaconu C.C., Nistor-Cseppento D.C. (2021). Efficiency of antiviral treatment in COVID-19. Exp. Ther. Med..

[7] Thanh Le T., Andreadakis Z., Kumar A., Gómez Román R., Tollefsen S., Saville M., Mayhew S. (2020). The COVID-19 vaccine development landscape. Nat. Rev. Drug Discov..

[8] WHO—COVID19 Vaccine Tracker. https://covid19.trackvaccines.org/agency/who/.

[9] Ritchie H., Ortiz-Ospina E., Beltekian D., Mathieu E., Hasell J., Macdonald B., Beltekian D., Roser M. (2020). Coronavirus Pandemic (COVID-19). https://ourworldindata.org/coronavirus.

[10] Coronavirus (COVID-19) Vaccinations. https://ourworldindata.org/covid-vaccinations.

[11] Goldhill O., Brodwin E., Cohrs R., Silverman E. (2021). Vaccination Rates Follow the Money in States with Big Wealth Gaps—STAT. https://www.statnews.com/2021/02/11/covid19-vaccination-rates-follow-the-money-in-states-with-biggest-wealth-gaps/.

[12] Magnan S. (2017). HealthPartners Institute Social Determinants of Health 101 for Health Care: Five Plus Five. NAM Perspect..

[13] Dalsania A.K., Fastiggi M.J., Kahlam A., Shah R., Patel K., Shiau S., Rokicki S., Dallapiazza M. (2021). The Relationship Between Social Determinants of Health and Racial Disparities in COVID-19 Mortality. J. Racial Ethn. Health Disparities.

[14] Hughes M.M., Wang A., Grossman M.K., Pun E., Whiteman A., Deng L., Hallisey E., Sharpe J.D., Ussery E.N., Stokley S. (2021). County-Level COVID-19 Vaccination Coverage and Social Vulnerability—United States, 14 December 2020–1 March 2021. MMWR. Morb. Mortal. Wkly. Rep..

[15] Puranik A., Venkatakrishnan A.J., Pawlowski C., Raghunathan B., Ramudu E., Lenehan P., Agarwal V., Jayaram S., Choudhary M., Soundararajan V. (2021). Higher COVID-19 vaccination rates are linked to decreased county-level COVID-19 incidence across USA. medRxiv.

[16] Caspi G., Dayan A., Eshal Y., Liverant-Taub S., Twig G., Shalit U., Lewis Y., Shina A., Caspi O. (2021). Socioeconomic Disparities and COVID-19 Vaccination Acceptance: Experience from Israel. Clin. Microbiol. Infect..

[17] Dror A.A., Eisenbach N., Taiber S., Morozov N.G., Mizrachi M., Zigron A., Srouji S., Sela E. (2020). Vaccine hesitancy: The next challenge in the fight against COVID-19. Eur. J. Epidemiol..

[18] Explore Health Rankings. https://www.countyhealthrankings.org/explore-health-rankings/rankings-data-documentation.

[19] Virtanen P., Gommers R., Oliphant T.E., Haberland M., Reddy T., Cournapeau D., Burovski E., Peterson P., Weckesser W., Bright J. (2020). SciPy 1.0: Fundamental algorithms for scientific computing in Python. Nat. Methods.

[20] Matplotlib: A 2D Graphics Environment. https://ieeexplore.ieee.org/document/4160265.

[21] Benjamini Y., Hochberg Y. (1995). Controlling the false discovery rate: A practical and powerful approach to multiple testing. J. R. Stat. Soc. Ser. B.

[22] Fernandez M.A.L., MA M. Delta Method in Epidemiology: An Applied and Reproducible Tutorial. https://migariane.github.io/DeltaMethodEpiTutorial.nb.html.

[23] Brown C.C., Young S.G., Pro G.C. (2021). COVID-19 vaccination rates vary by community vulnerability: A county-level analysis. Vaccine.

[24] (2021). COVID-19 Vaccination Provider Requirements and Support. https://www.cdc.gov/vaccines/covid-19/vaccination-provider-support.html#:~:text=COVID%2D19%20Vaccine%20is%20Provided%20at%20100%25%20No%20Cost%20to%20Recipients&text=Medicare%20or%20Medicaid%20reimbursement,-HRSA%20COVID%2D19.

[25] Patient Education Resources. https://www.aafp.org/family-physician/patient-care/current-hot-topics/recent-outbreaks/covid-19/covid-19-vaccine/patient-education-resources.html.

[26] Severe Housing Problems. https://www.countyhealthrankings.org/explore-health-rankings/measures-data-sources/county-health-rankings-model/health-factors/physical-environment/housing-transit/severe-housing-problems.

[27] Martins-Filho P.R. (2021). Relationship between population density and COVID-19 incidence and mortality estimates: A county-level analysis. J. Infect. Public Health.

[28] Stepanikova I., Oates G.R. (2017). Perceived Discrimination and Privilege in Health Care: The Role of Socioeconomic Status and Race. Am. J. Prev. Med..

[29] LaVeist T.A. (2005). Disentangling race and socioeconomic status: A key to understanding health inequalities. J. Urban. Health.

