# Supplementary material for: Counties with Lower Insurance Coverage and Housing Problems Are Associated with Both Slower Vaccine Rollout and Higher COVID-19 Incidence
Source: Vaccines (Basel). 2021 Aug 31;9(9):973. doi: 10.3390/vaccines9090973 (PMC8473044; doi:10.3390/vaccines9090973)
Supplement: Supplementary file 1 [file vaccines-09-00973-s001.zip › vaccines-1304086-supplementary.pdf]

Supplementary Materials

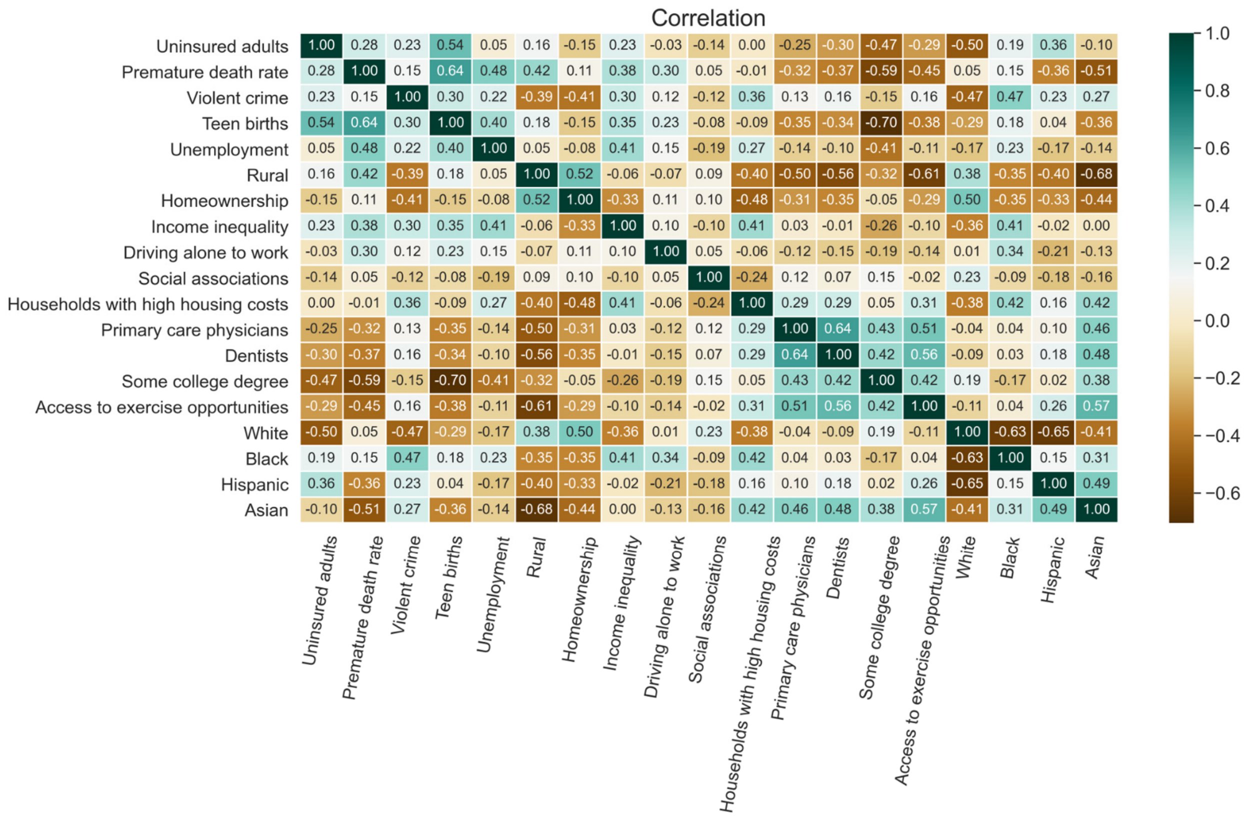

Figure S1. Heatmap of Spearman's rank correlation for selected county-level features. For each pair of features, Spearman rank correlation = +1 (**dark green**) corresponds to a perfectly monotonically increasing relationship, and Spearman rank correlation = -1 (**dark orange**) corresponds to a perfectly monotonically decreasing relationship. Correlation values are calculated based upon data available from the 3,087 counties in the study dataset, and features for each county include: demographic and socioeconomic variables, cumulative vaccinated percentage through June 6, 2021, and COVID-19 increase in incidence from December 1, 2020 to June 6, 2021.

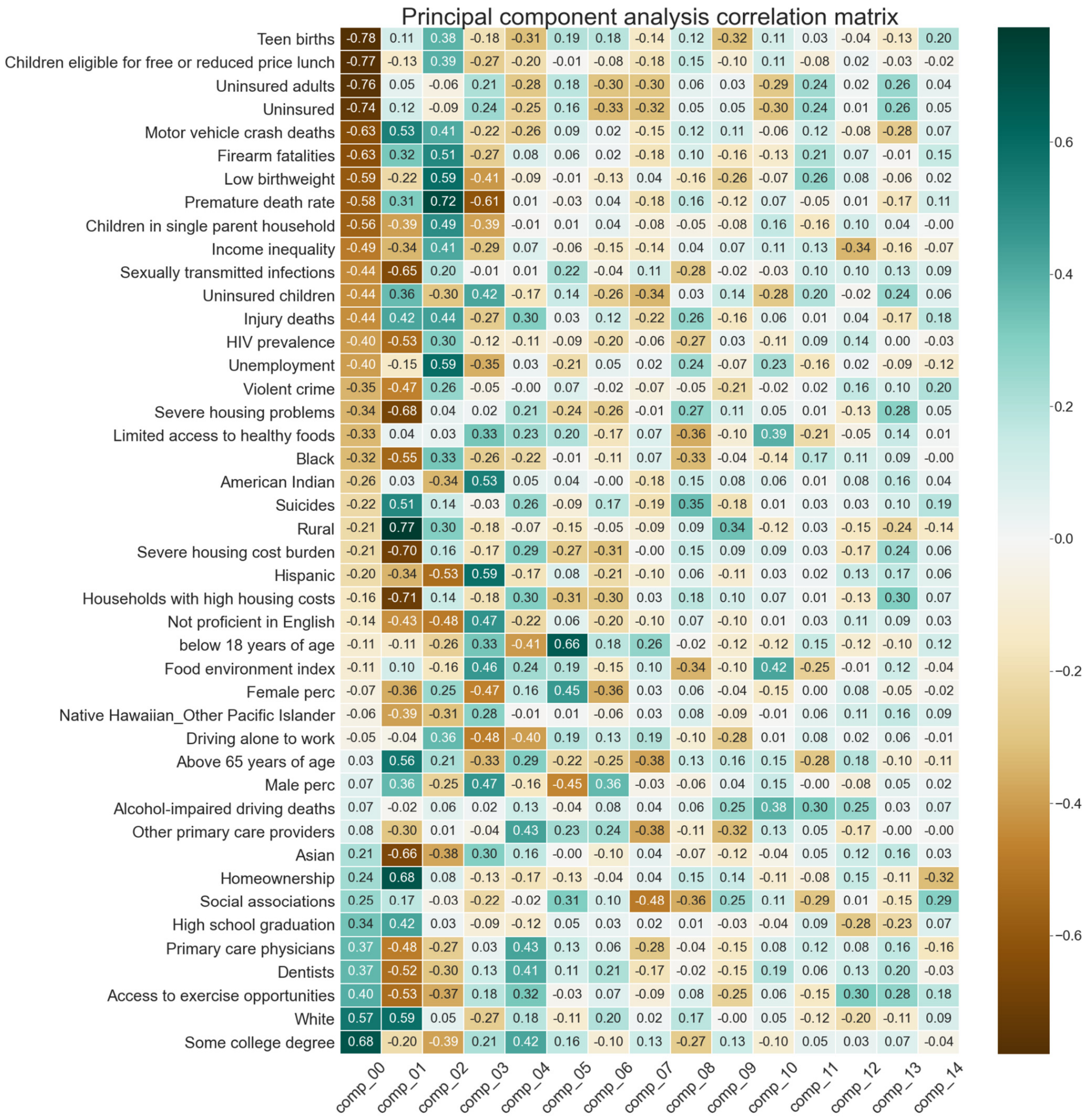

Figure S2: Correlation matrix between principal components and county-level features. The above correlation matrix shows correlation of each county-level feature with principal components. Rows correspond to socioeconomic features of the counties, and columns correspond to the principal components. In each cell, the Spearman rank correlation between the feature and the principal component is shown. The rows are sorted based upon the correlation with the first principal component, showing which features encode the most variability in the data.

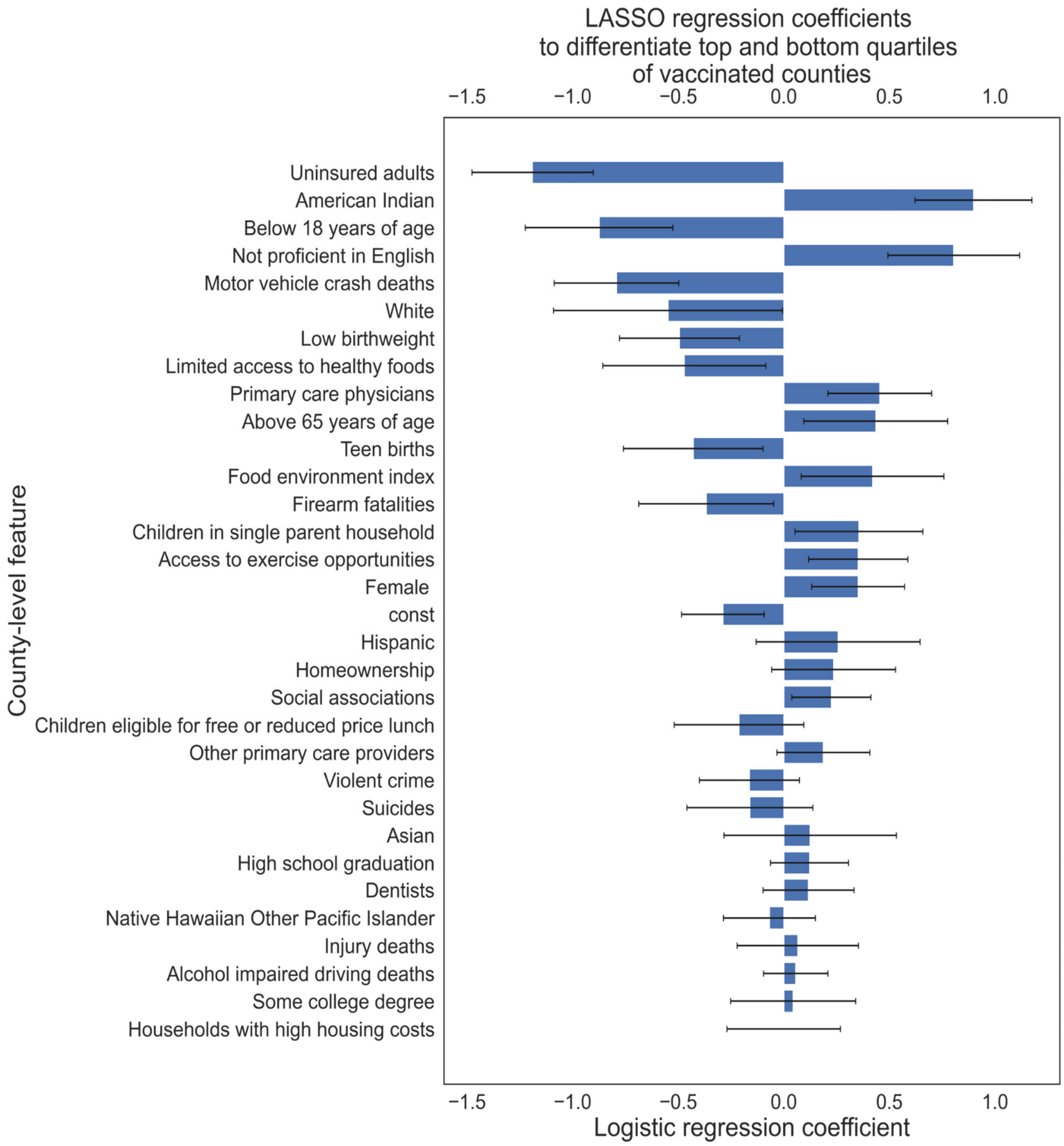

Figure S3: LASSO logistic regression coefficients and confidence intervals. The outcome variable for this LASSO logistic regression model is Y=1 if the county is in the top vaccinated quartile, and Y=0 if the county is in the bottom vaccinated quartile. All 44 socioeconomic factors were included as features for this LASSO model (normalized to have unit standard deviation), however only 31 were selected to have non-zero coefficients by the model, which are shown in the plot above. For each of these features, the magnitude of the coefficient is shown along with error bars indicating the 95% confidence interval.

Table S1. Top 25 US Counties ranked by percent of uninsured population. Information on state, county, percent uninsured, percent vaccinated, increase in COVID-incidence per 100,000 people as of June 6, 2021 relative to December 1, 2020, percent rural, population and percent male are provided for each county.

Uninsured Rank	State	County	% Uninsured	% Vaccinated	COVID incidence increase (per 100k) from Dec 1 to Jun 6	% Rural	Population	% Male
1	Texas	Gaines County	33.7	13.5	-181.5	63.0	21,492	50.6
2	Texas	Collingsworth County	32.7	24.2	-68.5	100.0	2,920	49.0
3	Texas	Briscoe County	31.4	29.1	-64.7	100.0	1,546	49.7
4	Texas	Hidalgo County	31.2	44.0	-203.2	5.1	868,707	49.0
5	Texas	Dallam County	30.5	26.8	-13.7	23.5	7,287	52.6
6	Texas	Sherman County	29.6	22.9	N/A	100.0	3,022	52.2
7	Texas	Lipscomb County	29.5	22.9	-402.1	100.0	3,233	51.1
8	Texas	Jeff Davis County	29.4	42.9	-1231.3	100.0	2,274	51.2
9	Texas	Zapata County	29.2	41.0	-550.1	23.5	14,179	49.7
10	Texas	Presidio County	29.0	63.6	-2759.5	40.5	6,704	51.3
11	Texas	Cameron County	29.0	46.2	-90.7	8.4	423,163	48.8
12	Texas	San Saba County	28.9	21.9	-181.7	49.4	6,055	54.2
13	Texas	Starr County	28.9	51.1	-111.4	23.7	64,633	48.8
14	Texas	Castro County	28.9	25.2	-252.3	45.8	7,530	51.3
15	Texas	Parmer County	28.7	27.9	-270.7	60.0	9,605	51.7
16	Texas	Ochiltree County	28.6	27.6	-569.3	13.9	9,836	50.2
17	Texas	Webb County	28.6	48.4	-388.9	2.6	276,652	49.2

18	Texas	Hudspeth County	28.5	45.9	-2005.7	100.0	4,886	53.1
19	Texas	Cochran County	28.5	27.1	-35.1	100.0	2,853	50.7
20	Texas	Mason County	28.1	35.4	117.0	100.0	4,274	51.1
21	Alaska	Aleutians East Borough	27.9	63.2	-329.6	100.0	3,337	67.5
22	Texas	Terrell County	27.7	38.4	-1546.4	100.0	776	50.4
23	Texas	Moore County	27.7	29.3	-191.0	16.8	20,940	51.9
24	Georgia	Echols County	27.6	18.2	-249.6	100.0	4,006	51.8
25	Texas	Cottle County	27.4	28.6	-286.1	100.0	1,398	48.7

Table S2: LASSO logistic regression coefficients to differentiate top and bottom quartile vaccinated counties. The outcome variable for this LASSO logistic regression model is Y=1 if the county is in the top vaccinated quartile, and Y=0 if the county is in the bottom vaccinated quartile. All 44 socioeconomic factors were included as features for this LASSO model (normalized to have unit standard deviation), however only 31 were selected to have non-zero coefficients by the model, which are presented in the table below. For each of these features, the following are presented: (1) coefficient of the feature in the logistic regression model along with the associated 95% confidence interval (CI), (2) Odds ratio of the feature in the logistic regression model along with the associated 95% CI, and (3) p-value for the feature in the logistic regression model. Rows are sorted by coefficients of regression.

County-level feature	Coefficient (95% CI)	Odds ratio (95% CI)	p-value
Uninsured adults	-1.190 (-1.477, -0.902)	0.304 (0.228, 0.406)	<0.001
Below 18 years of age	-0.874 (-1.224, -0.525)	0.417 (0.294, 0.592)	<0.001
Motor vehicle crash deaths	-0.792 (-1.087, -0.498)	0.453 (0.337, 0.608)	<0.001
White	-0.548 (-1.091, -0.006)	0.578 (0.336, 0.994)	0.048
Low birthweight	-0.494 (-0.778, -0.210)	0.610 (0.459, 0.811)	0.001
Limited access to healthy foods	-0.471 (-0.857, -0.085)	0.624 (0.424, 0.919)	0.017
Teen births	-0.429 (-0.760, -0.098)	0.651 (0.468, 0.907)	0.011
Firearm fatalities	-0.367 (-0.687, -0.048)	0.693 (0.503, 0.953)	0.024
const	-0.288 (-0.484, -0.093)	0.750 (0.616, 0.911)	0.004
Children eligible for free or reduced price lunch	-0.213 (-0.520, 0.094)	0.808 (0.595, 1.099)	0.174
Violent crime	-0.163 (-0.400, 0.074)	0.850 (0.670, 1.077)	0.179
Suicides	-0.161 (-0.459, 0.137)	0.851 (0.632, 1.147)	0.29
Native Hawaiian Other Pacific Islander	-0.068 (-0.286, 0.149)	0.934 (0.751, 1.161)	0.537
Households with high housing costs	-0.001 (-0.269, 0.268)	0.999 (0.764, 1.307)	0.997
Some college degree	0.044 (-0.252, 0.340)	1.045 (0.777, 1.405)	0.771
Alcohol impaired driving deaths	0.056 (-0.096, 0.209)	1.058 (0.908, 1.232)	0.468

Injury deaths	0.066 (-0.221, 0.353)	1.068 (0.802, 1.423)	0.652
Dentists	0.116 (-0.099, 0.332)	1.123 (0.906, 1.394)	0.29
High school graduation	0.122 (-0.063, 0.306)	1.13 (0.939, 1.358)	0.197
Asian	0.124 (-0.284, 0.533)	1.132 (0.753, 1.704)	0.55
Other primary care providers	0.187 (-0.033, 0.407)	1.206 (0.968, 1.502)	0.096
Social associations	0.225 (0.038, 0.412)	1.252 (1.039, 1.510)	0.019
Homeownership	0.236 (-0.058, 0.529)	1.266 (0.944, 1.697)	0.116
Hispanic	0.257 (-0.131, 0.645)	1.293 (0.877, 1.906)	0.194
Female	0.352 (0.132, 0.571)	1.422 (1.141, 1.770)	0.002
Access to exercise opportunities	0.352 (0.117, 0.587)	1.422 (1.124, 1.799)	0.003
Children in single parent household	0.355 (0.053, 0.658)	1.426 (1.054, 1.931)	0.021
Food environment index	0.420 (0.083, 0.758)	1.522 (1.087, 2.134)	0.015
Above 65 years of age	0.435 (0.095, 0.775)	1.545 (1.100, 2.171)	0.012
Primary care physicians	0.454 (0.209, 0.700)	1.575 (1.232, 2.014)	<0.001
Not proficient in English	0.804 (0.492, 1.116)	2.234 (1.636, 3.053)	<0.001
American Indian	0.898 (0.621, 1.174)	2.455 (1.861, 3.235)	<0.001
